# High-throughput screening of small molecules targeting *Mycobacterium tuberculosis* in human iPSC macrophages

**DOI:** 10.1128/aac.01613-24

**Published:** 2025-05-27

**Authors:** Alejandro Armesilla-Diaz, María Pilar Arenaz, Charlotte Ashby, Delia Blanco, Emiliana D'Oria, Helena Garuti, Vanesa Gómez, Rubén González-Del-Río, María Martínez-Hoyos, Eugenia Meiler, Alfonso Mendoza-Losana, Lisa Mohamet, Laura Padrón-Barthe, Esther Pérez, Laura Pérez, Modesto J. Remuiñán, Beatriz Rodríguez-Miquel, Delfina Segura-Carro, Sara Viera-Morilla

**Affiliations:** 1 Genomics Sciences, GlaxoSmithKline, Stevenage, United Kingdom; 2 Global Health Medicines R&D, GlaxoSmithKline, Madrid, Spain; 3 MMD-MD, GlaxoSmithKline, Madrid, Spain; St. George's, University of London, London, United Kingdom

**Keywords:** tuberculosis, drug screening, host-pathogen interactions, human induced pluripotent stem cells, macrophages

## Abstract

New treatments are still necessary to eradicate tuberculosis disease. Macrophages derived from human induced pluripotent stem cells (hiPSC-Macs) offer a physiological niche to identify potential new drugs in the context of *Mycobacterium tuberculosis* (Mtb) infection. Here, we describe the scale-up of hiPSC-Macs production in 5-stack chambers for high-throughput drug screening against Mtb. A rate of approximately 100 million hiPSC-Macs was generated with optimal quality for a period of up to 12 weeks. Moreover, the infection model was optimized using a luminescence-based Mtb reporter strain. The assay showed enough sensitivity to identify compounds that could target host-pathogen interactions during Mtb infection. We interrogated a library of 200,000 compounds in Mtb-infected hiPSC-Macs with a Z-score above 0.3 in all plates analyzed. After secondary assays, 223 qualified hits were selected for further progression.

## INTRODUCTION

Tuberculosis (TB) is the leading cause of death from a single infectious agent, *Mycobacterium tuberculosis* (Mtb); except for the 2020–2022 period, when it was surpassed by the global pandemic of the coronavirus, severe acute respiratory syndrome coronavirus 2 (SARS-CoV-2). The current regimen for drug-susceptible TB consists of isoniazid, rifampicin, pyrazinamide, and ethambutol. Despite the efficacy of this combination therapy, the extended treatment duration and numerous side effects negatively impact patient adherence. This, in turn, contributes to the emergence of drug-resistant TB. To achieve the goal of “The End of TB Strategy” proposed by the World Health Organization by 2035 (1), there is an urgent need to develop safer regimens with improved efficacy and shorter treatment duration.

Adjunctive therapies targeting either Mtb virulence factors or host immune response have been proposed as promising strategies to tackle the selection of resistant strains while reducing the pathology of the disease (2, 3). In fact, corticosteroids are already administered in conjunction with standard-of-care therapies for TB meningitis and for prevention and treatment of paradoxical reactions in patients co-infected with HIV and Mtb (4, 5). However, the number of preclinical programs targeting host-pathogen interactions within the TB pipeline remains limited, with most clinical trials focused on the repurposing of host-directed therapies (HDTs) for pulmonary TB (6). This gap is likely due to the challenges associated with the identification of new TB HDTs, the absence of a well-defined progression cascade, and the lack of suitable models for the development of such therapeutics.

During the last decade, several intracellular high-throughput screens (HTS) have been performed to identify compounds targeting host-pathogen interactions (HPI) during Mtb infection in different cell types, such as human fibroblasts (7, 8). Macrophages (Macs) are the first host cell targets for Mtb infection and play an essential role in disease progression (9). Extensive studies have utilized Raw264.7 and J774 murine Macs or the human monocytic THP-1 cell line as a cost-effective means to perform Mtb intracellular drug screening (10–13). However, a growing body of evidence demonstrates their limited translational relevance to mimicking either human immune responses during Mtb infection or the ability to reproduce human primary peripheral blood mononuclear cell macrophages (hPBMC-Macs) or alveolar macrophages at both functional and molecular levels (14). Consequently, this leads to identifying compounds that are targeting non-relevant mechanisms in humans and thus compromising the discovery of clinically efficacious compounds targeting HPI (13, 15, 16). Although primary human alveolar macrophages and hPBMC-Macs would be the preferred physiologically relevant *in vitro* models to study human HPI, they cannot be cultured at a sufficient scale to enable HTS campaigns.

Significantly, human induced pluripotent stem cell macrophages (hiPSC-Macs) have been recently described as a valid model for mycobacterial infection (17, 18) and for non-infectious pulmonary diseases (19, 20). hiPSC-Macs could facilitate the discovery of the mechanisms that drive activation, tissue-specific phenotypes, function, and dysfunction in disease. This cellular model retains plasticity and can be polarized using stimuli to express canonical markers of pro- and anti-inflammation consistent with hPBMCs and tissue-resident macrophages (21–23). Furthermore, extensive transcriptional profiling of *in vitro* macrophage models demonstrates substantial overlap between hPBMCs and hiPSC-Macs (24–26). Along with the ability to have an infinite source of iPSCs, these characteristics show that hiPSC-Macs are a more physiologically relevant and scalable model compared to current immortalized cell lines for identifying novel compounds and combinations that might better correlate with a successful clinical outcome.

Despite this, there have been few publications to date describing the use of hiPSC-Macs in HTS for drug discovery. This could be likely attributed to both the cost of the production of hiPSC-Macs and the expertise required to ensure the robust scaling of the model. Here, we describe a method for generating and characterizing hiPSC-Macs at a screening scale and demonstrate a robust infection assay with sufficient sensitivity to detect compounds targeting HPI. We performed HTS to interrogate a library of 200,000 compounds using a luminescence-based reporter assay to identify novel small molecules targeting HPI during Mtb infection in hiPSC-Macs. We identified 223 compounds with the desired hit profile to validate further as potential novel HPI candidates for future drug optimization programs.

## MATERIALS AND METHODS

### Generation of human peripheral blood mononuclear cells derived macrophages

All donors provided written informed consent for the use of their samples, and the collection and use of samples received Institutional Review Board or Ethics Committee approval (CEI-131-2709). Buffy coats from healthy blood donors were anonymously provided by the Comunidad de Madrid blood bank. Ethical approvals for all blood sources and processes used in this study were approved by the GSK Ethics Committee. All experiments were carried out in accordance with the approved guidelines and regulations.

The hPBMCs were isolated from buffy coats through a Ficoll density gradient centrifugation. The buffy coat was split into 15 mL sterile tubes containing 15 mL of calcium and magnesium-free Dulbecco's phosphate-buffered saline (DPBS). This mix was carefully added to a Falcon tube containing 15 mL of Ficoll-Paque-1.077 g/mL (Cytiva). Centrifugation was done at 500 × *g* for 30 minutes and 21°C with the brake off. The mononuclear cells were collected from the plasma/Ficoll interface in a new Falcon filled with DPBS. Cells were washed three times by centrifugation at 300 × *g* for 10 minutes at 4°C. Platelets were removed by an additional centrifugation at 200 × *g* for 10 minutes. Cells were resuspended in DPBS, counted with a NucleoCounter NC-202 (Chemometec), and resuspended in the appropriate MACS buffer (Miltenyi) volume, according to the manufacturer’s protocol.

CD14-positive monocytes were isolated by a magnetic positive selection using anti-human CD14 microbeads (Miltenyi). Purified monocytes were washed and resuspended in RPMI-1640 GlutaMAX supplemented with 10% FBS and 100 ng/mL M-CSF (Peprotech). Finally, they were seeded in non-treated culture flasks at a density of approximately 60,000 cells/cm^2^ and incubated at 37°C with 5% CO_2_ and humidity for 5–7 days for monocyte-derived macrophage differentiation.

### Generation of human iPSC-derived macrophages

Human iPSC lines 66540088 (WTSli018A) and 66540004 (UKBi006A) were supplied by the European Bank for Induced Pluripotent Stem Cells (EBiSC), and the use of samples received Ethics Committee approval (CEI-117-2357). Frozen stocks of hiPSC were expanded in Vitronectin (STEMCELL Technologies) coated flasks and serum-free conditions using Essential 8 (E8) FLEX medium (Gibco). Cells were cultured at 37°C with humidity and 5% CO_2_. The medium was changed daily, and cells were passaged once at 70%–80% confluency using ReleSR (STEMCELL Technologies). We performed karyotype analysis on all hiPSC lines at passages 1 and 6 upon receipt from EBiSC using both G-banding and comparative hybridization. All hiPSCs had a normal karyotype as detected using G-banding. However, comparative hybridization detected a small duplication on the long arm of chromosome 20 that includes the BCL2L1 gene, a common mutation in iPSCs, in WTSli018A at passage 1. Nevertheless, in passage 6, no additional karyotypical abnormalities were detected.

Embryoid bodies (EB) were formed using AggreWell technology. AggreWell 800 plates (STEMCELL Technologies) were prepared by rinsing with 500 µL/well of anti-adherence rinsing solution (STEMCELL Technologies), centrifuged at 312 × *g* for 5 minutes, and observed using an inverted microscope to ensure no bubbles remained in the microwells. Wells were washed with E8 media and 1 mL/well of E8 supplemented with 10 µM ROCK inhibitor (STEMCELL Technologies), 50 ng/mL BMP-4 (R&D Systems), 20 ng/mL SCF (R&D Systems), and 50 ng/mL VEGF (R&D Systems). hiPSCs were washed with DPBS, detached with Versene (Gibco), and centrifuged at 240 × *g* for 5 minutes before being resuspended in E8 medium supplemented as described above. A single cell suspension was generated by gently pipetting with a serological pipette, and 1 mL of the cellular suspension (1.5 × 10^6^ cells/mL) was added to each well of the AggreWell plate already containing 1 mL of complete E8 media. Cells were captured in the microwells by centrifugation at 100 × *g* for 3 minutes. The AggreWell plates were maintained at 37°C with 5% CO_2_ and humidity for 4 days. Each day, EBs’ culture medium was refreshed by removing 1 mL/well of media and adding 1 mL/well of fresh E8 media supplemented as described above without ROCK inhibitor. On day 4, the contents of the microwells were collected in a Falcon tube by gently pipetting up and down with fresh media. The EBs were then resuspended in 500 mL of XVIVO-15 medium (Lonza) supplemented with 25 ng/mL IL-3 (Peprotech), 100 ng/mL M-CSF (Peprotech), 2 mM GlutaMAX (Gibco), and 55 µM β-mercaptoethanol (Gibco) and transferred to gelatin-treated 5-stacks (Corning) for continuous hiPSC-monocyte-like production.

Once in the 5-stack chambers, EBs adhered to the culture surface with the resultant hiPSC-monocyte-like cells found in the supernatant. Non-adherent hiPSC-monocyte-like cells were harvested every 7–8 days, referred to as a “lot,” and the EBs were maintained by replacing half of the media. Following 2–3 weeks, approximately 100 million hiPSC-monocyte-like cells per week were produced, and 500 mL of the supernatant was replaced with fresh media. The EB monolayers were maintained for 12 weeks or longer.

Collected hiPSC-monocyte-like cells from lot three onward were centrifuged at 480 × *g* for 15 minutes and resuspended in fresh macrophage media (RPMI-1640 GlutaMAX supplemented with 10% FBS and 100 ng/mL M-CSF). Finally, they were seeded in non-treated culture flasks at a density of *ca*. 50,000 cells/cm^2^ and incubated at 37°C with 5% CO_2_ and humidity for 5–7 days to induce macrophage differentiation.

### Flow cytometry

As a quality control of hiPSC-Macs production, several hematopoietic and canonical macrophage surface markers were assessed using human-specific antibodies: CD14-APC (eBiosciences), CD11b-APC (eBiosciences), CD45-Alexa488 (Biolegend), CD86-APC (Biolegend), CD163-FITC (R&D), and CD206-APC (Biolegend). IgG1-488 and IgG2b-APC (Biolegend) isotype controls were included. Monocytes or macrophages were blocked with TruStain FxC (Biolegend) and stained with the specific fluorochrome-conjugated antibodies for 30 minutes. After washing, the cells were analyzed using the BD LSR II flow cytometer, and data were analyzed using FlowJo X software. This was performed on differentiated macrophages on the first and last lots used for intracellular assays of every production.

### Phagocytosis

hiPSC monocyte-like cells were seeded at 3,500 cells/well (384-well plate) in 100 ng/mL M-CSF (Peprotech) for 6 days to terminally differentiate into macrophages. pHrodo-NTHi particles (generated in-house) were resuspended to 1 mg/mL in assay buffer, sonicated for 2 minutes, and then passed through a 23 g blunt needle. A 1 mL pHrodo was added to 3 mL assay buffer to achieve a 0.25 mg/mL assay stock. To allow phagocytosis to proceed, pHrodo assay stock was added to the macrophages, and the plate was incubated for 3 hours at 37°C, 5% CO_2_. Subsequently, samples were fixed and stained with Hoechst (Invitrogen). Images were acquired using the INCell Analyzer 2200, and analysis was performed in the Columbus system (PerkinElmer).

### Mycobacterial strains and plasmid construction

Mtb H37Rv (ATCC 25618) strain, expressing pMV361 NanoLuc Luciferase reporter plasmid, was utilized for all experiments. The strain was routinely grown at 37°C in 7H9 medium supplemented with 0.2% glycerol, Middlebrook ADC enrichment (0.5% bovine albumin fraction, 0.2% dextrose, 0.003% catalase), and 0.025% tyloxapol.

The integrative luciferase assay vector pMV361 NanoLuc was obtained using pMV361 as a backbone, a mycobacterial shuttle plasmid containing hps60 promoter upstream of a multi-cloning site and a kanamycin marker. pNL1.1.CMW (Promega) was used to amplify the NanoLuc sequence, and *EcoRI* and *ClaI* restriction sites were introduced by PCR with the primers 5′-CACAGTTGAATTCCTAACGCAGTCAGTGG-3′ and 5′-CAATGTATCTTATCATGTCTGATCGATGCGGCC-3′. The fragment was double digested, and the derived 611 bp-long fragment was ligated into a pMV361 vector previously linearized by *EcoRI* and *ClaI* digestion. The final plasmid, pMV361 NanoLuc, was transformed into *E. coli* DH5α, and recombinant strains were verified by Sanger sequencing. The Mtb H37Rv pMV361 NanoLuc strain was made by electroporating electrocompetent Mtb H37Rv (ATCC 25618) with the pMV361 NanoLuc plasmid and plating on selective media. Positive clones were selected with kanamycin and confirmed by luminescence.

To establish the correlation between the number of bacteria and light emission, we performed serial dilutions (series of 23 dilutions, factor ½, by duplicate in a 384-well plate) to determine actual colony-forming units (CFUs). Light emission was measured using the Promega Nano-Glo Luciferase Assay System. A 170 minute kinetic curve was made, and R^2^ was calculated using the relative light units (RLUs) at the time of maximum emission (40 minutes).

To measure extracellular activity of the compounds, an inoculum of Mtb H37Rv pMV361 NanoLuc strain was prepared at 100,000 CFUs/mL, and 50 µL/well was dispensed in 384-well plates. After 5 days of incubation, light emission was measured using the Promega Nano-Glo Luciferase Assay System with an EnVision Multilabel Plate Reader (PerkinElmer).

### Bacterial culture preparation for intracellular assay

Mtb H37Rv cultures were grown to the exponential phase, and bacteria were pelleted and washed with DPBS at 3,500 rpm for 10 minutes. An equal volume of sterile glass beads (3.5 mm) that matched the pellet size was added and then vigorously shaken for 2 minutes to break up bacterial clumps. The bacteria were rewashed with DPBS at 1,200 rpm for 5 minutes. The supernatant was transferred into a new tube, and optical density at 600 nm was measured. Glycerol was added to a final concentration of 5% to the supernatant, and 100 µL aliquots were frozen at −80°C for up to 6 months. Actual CFU number was estimated after thawing by plating 50 µL of 10-fold serial dilutions from the bacterial suspension in 7H10 supplemented with Middlebrook OADC (Oleic acid, Albumin, Dextrose, and Catalase) and 0.2% glycerol plates. CFU counting was assessed after incubation for 2–3 weeks at 37°C and 5% CO_2_.

### Intracellular TB assay

hiPSC (or hPBMC)-Macs were infected at a multiplicity of infection (MOI) of 1. The required volume of the single-cell mycobacterial stock described above was thawed in the macrophage media and incubated overnight at 37°C. The infection was done by adding the bacterial suspension to the flasks containing the macrophages. After 20 hours, infected cells were washed five times to remove the extracellular bacteria and detached with Versene for 30 minutes (Gibco). Once detached, cells were washed twice and resuspended in fresh macrophage media. Infected cells were finally resuspended (1.5 × 10^5^ cells/mL or 1 × 10^5^ cells/mL for hiPSC-Macs or hPBMC-Macs, respectively) in complete media. Subsequently, 50 µL of cell suspension was dispensed per well in white, flat-bottom 384-well plates (Greiner). Plates were incubated for 5 days at 37°C with 5% CO_2_ and humidity. After incubation, plates were revealed using the Nano-Glo Luciferase Assay System (Promega), and end-point luminescence was measured with an EnVision Multilabel Plate Reader (PerkinElmer).

To ensure experiment reliability, we used internal controls: 16 wells containing DMSO (at a concentration of 0.5%) as the maximum light signal reference, and 16 wells containing moxifloxacin (at a 20 µM final concentration), representing the minimal light signal reference. This allowed us to establish upper and lower luminescence limits, respectively. These were used to perform the statistical analysis by calculating the Z-score (27). In addition, they were used to calculate the compounds' percentage of inhibition using Activity Base XE. In dose-response assays, curves were plotted by representing the percentage of inhibition versus the compound’s log concentration using TIBCO Spotfire. The inhibitory concentration of compounds able to kill 50% (IC50) of bacteria was interpolated from the fitted curve equation.

### Robustness set

A subset of 1,405 small molecules from the GSK HTS collection was tested at a final concentration of 10 µM in 0.5% DMSO.

### Chemical synthesis of the tool compounds

Compounds were obtained from the GSK compound collection or prepared following published methodologies (7) (WO/2016/041972).

### Statistical analysis

Statistical analysis was performed using one-way analysis of variance (ANOVA) or unpaired Student’s *t*-test; *P* < 0.05 and below were considered significantly different (*P < 0.05, **P < 0.01, ***P < 0.001, and ****P < 0.0001).

## RESULTS

### Generation and characterization of hiPSC-Macs

**Fig 1:**
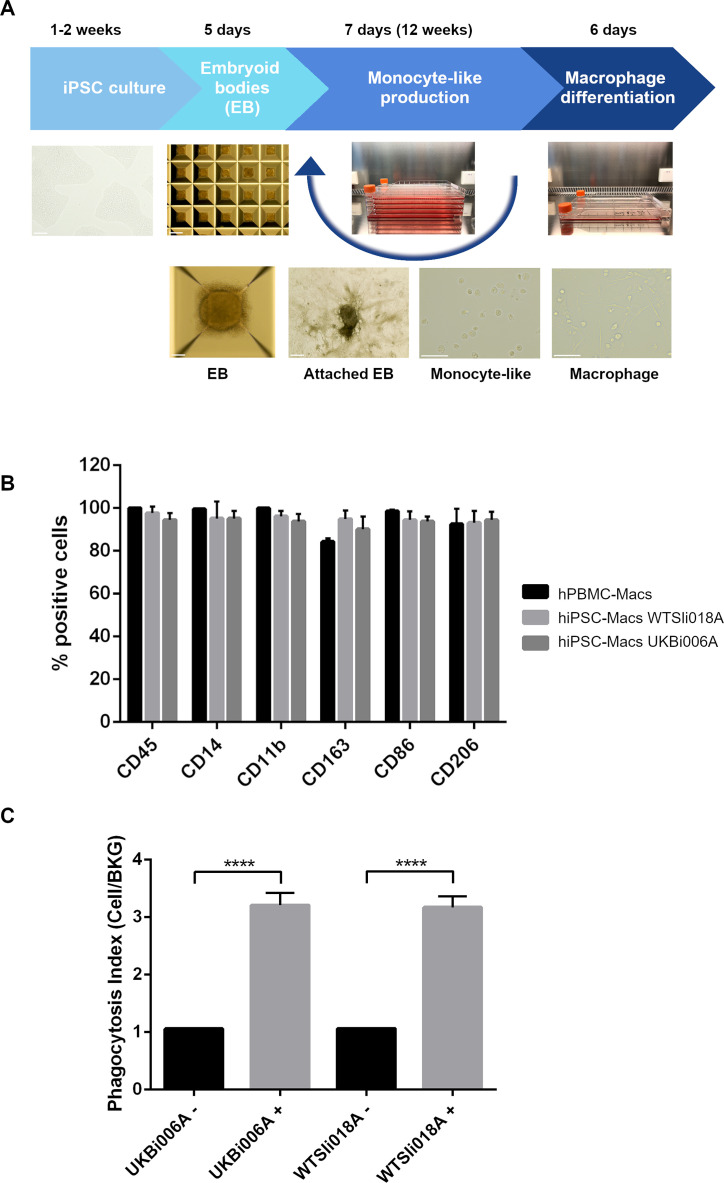
(A) Schematic diagram of hiPSC (WTSIi018A) myeloid lineage commitment stages from undifferentiated hiPSCs to macrophages using a method adapted from van Wilgenburg et al. (28). There are four key lineage commitment stages during hiPSC-Mac differentiation: mesoderm and hemogenic endothelium induction (EB and EB attachment stage); hematopoiesis and myeloid specification (monocyte-like production); and terminal differentiation to hiPSC-Macs. We utilized AggreWells for EB formation and 5-stack flasks to generate approximately 100 million hiPSC-monocyte-like cells per week over a 12 week period. Representative phase-contrast images of each differentiation step are shown. Pictures were taken with Evos XL Core (AMEX1000), hiPSC pictures were taken at 20×, EB and EB attached pictures were taken at 4×, monocyte-like and hiPSC-Macs were taken at 40×. The scale bar represents 500 µm for the EB plate panel and 100 µm for the rest of the panels. (B) Flow cytometry analysis of macrophage markers present in hiPSC-Macs differentiated from WTSIi018A and UKBi006A donors and from peripheral blood mononuclear cells derived macrophages (hPBMCs-Macs). hiPSC-Macs express pan-macrophage lineage markers in >80% of the cell population and show expression patterns similar to hPBMC-Macs. Means derived from three replicates of hiPSC-Macs (WTSIi018A and UKBi006A) and three donors from hPBMC-Macs; error bars represent ±SD. No statistically significant differences were observed when comparing the three groups using one-way ANOVA. (C) Functional assessment of hiPSC-Macs phagocytic activity. Phagocytosis levels were determined following incubation with (+) or without (–) pHrodo-labeled bacteria. Data were derived from two different donors of hiPSC-Macs (WTSIi018A and UKBi006A, *n* = 16 per donor). The phagocytosis index corresponds to the pHrodo Red cell signal normalized to background fluorescence. Error bars represent ±SD. The difference between groups was statistically significant based on Students’ unpaired *t*-test analysis; ****, *P* < 0.0001.

hiPSC-Macs from two different donors (WTSIi018A and UKBi006A) were generated from EBs in a feeder-free medium adapted from van Wilgenburg et al. (28) (Fig. 1A for schematic diagram). Prior to every experiment, hiPSC-Macs and hPBMC-Macs were assessed for the expression of a panel of myeloid lineage markers using flow cytometry following 6 days of culture in M-CSF (Fig. 1B). hiPSC-Macs expressed macrophage markers concomitant to hPBMC-Macs with no statistically significant differences. For inclusion in all subsequent experiments, hiPSC-Macs were required to express CD14, CD45, CD11b, CD86, CD163, and CD206 in >80% of the cell population. We further validated the functionality of hiPSC-Macs by employing a phagocytosis assay using pHrodo-NTHi particles (Fig. 1C). hiPSC-Macs retained robust phagocytic activity, as previously reported (28).

### Mtb infection of hiPSC-Macs and hPBMC-Macs

**Fig 2:**
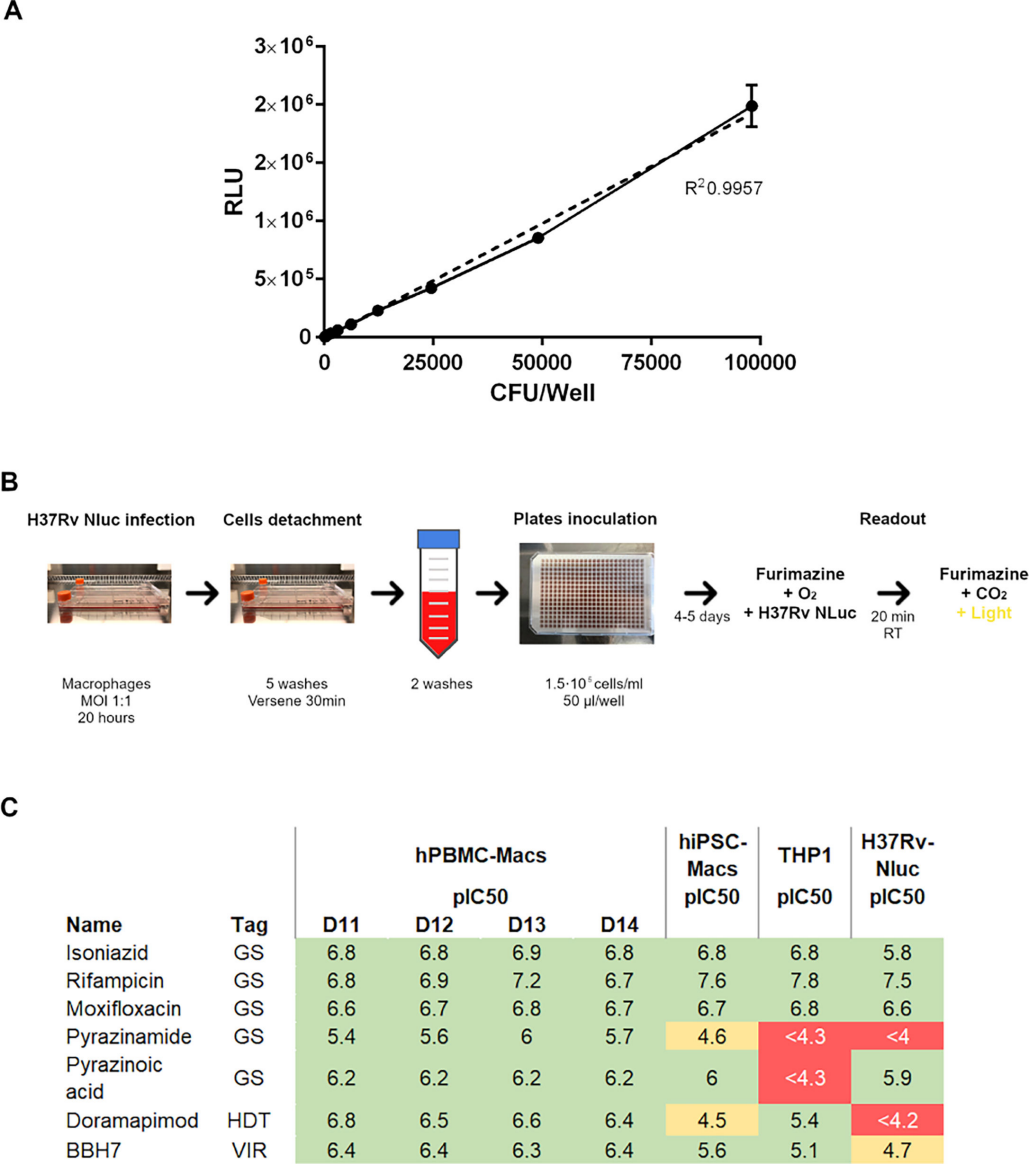
(A) Correlation between CFUs and RLUs in H37Rv NLuc reporter strain (R^2^ = 0.9957). (B) Schematic view of hiPSC-Macs and hPBMC-Macs infection. RT, Room Temperature. (C) pIC50 of tool compounds in intracellular (H37Rv-infected hPBMC-macs, hiPSC-macs, and THP1 cells) and extracellular (H37Rv-NLuc) assays. GS, Gold Standard; HDT, Host-Directed Therapy; VIR, virulence inhibitor.

Based on the data above, we decided to set up an intracellular Mtb assay using hiPSC-Macs in a high-throughput format to identify new molecules that interfere with relevant processes during macrophage infection. To increase the sensitivity of the assay, we designed a construct encoding a NanoLuc (NLuc) reporter (Promega) under the control of the hsp60 promoter in an integrative plasmid for its constitutive expression in mycobacterial species. This reporter strain showed higher sensitivity than the one obtained with a luciferase reporter (29). Importantly, we obtained a good correlation between CFU and RLUs for H37Rv NLuc reporter strain (Fig. 2A).

hiPSC-Macs or hPBMC-Macs were differentiated into macrophages for 5–7 days and infected at an MOI of 1 with H37Rv NLuc for 20 hours. Then, the bacterial growth was monitored over time, and the endpoints of the assays were selected (at day 5 of infection for hiPSC-Macs and day 4 for hPBMC-Macs) following RLU. During the infection setup, we observed a higher signal-to-background ratio in Mtb-infected hiPSC-Macs derived from hiPSC donor WTSIi018A compared to UKBi006A, similar to hPBMC-Macs (Fig. S1). Furthermore, we found that the infection of hiPSC-Macs from donor WTSIi018A showed less variability in the internal controls when compared with hiPSC donor UKBi006A, contributing to Z-scores >0.3, which was used as a cut-off. We, therefore, decided to go on the screening with the WTSIi018A donor.

Once we established the conditions of the infection, the monocyte production and differentiation from donor WTSIi018A were scaled up to improve the robustness of the assay (Fig. 2B), adapting the protocol previously described by van Wilgenburg et al. (28). The results demonstrated that the production of monocyte-like cells could be maintained at a weekly rate of approximately 100 million cells for a period of up to 12 weeks.

Once the conditions of the assays were established, we compared the *in vitro* activity of compounds in Mtb-infected hiPSC-Macs, hPBMC-Macs, and THP-1 cells (12). The aforementioned cell types were infected with the H37Rv NLuc reporter strain, and the pIC50 {pIC50 = −log_10_[IC50 (M)]} was evaluated with a set of gold-standard antibiotics for TB and tool compounds previously described to be involved in HPI (Fig. 2C [7]). The extracellular activity of this set of compounds was also determined using the H37Rv NLuc reporter strain. Moxifloxacin was used as an inhibitory control, and DMSO as a growth control. Most classical antibiotics used in the first-line and second-line TB treatment showed similar results between THP1, hiPSC-Macs, hPBMC-Macs, and extracellular assays. Exceptionally, pyrazinamide (PZA) and pyrazinoic acid showed activity in hiPSC-Macs and hPBMC-Macs, but both compounds were inactive in THP1 cells.

Some compounds previously described to inhibit virulence mechanisms, such as BBH7 (7), were consistently more active in hiPSC-Macs than in our routine THP1 assay (Fig. 2C). This underscores that the hiPSC-Macs assay could enhance the detection of this kind of inhibitors in an HTS due to its ability to closely mimic human macrophage behaviour. However, the pIC50 of HDT, such as doramapimod, showed discrepancies in activity between these Mtb intracellular models that could be due to differential expression of the target in the aforementioned cell types. In summary, these results reflect that the model based on hiPSC-Macs could potentially select classical antibiotics and compounds targeting HPI not previously identified in past screening campaigns performed with the GSK library.

### Robustness test

**Fig 3:**
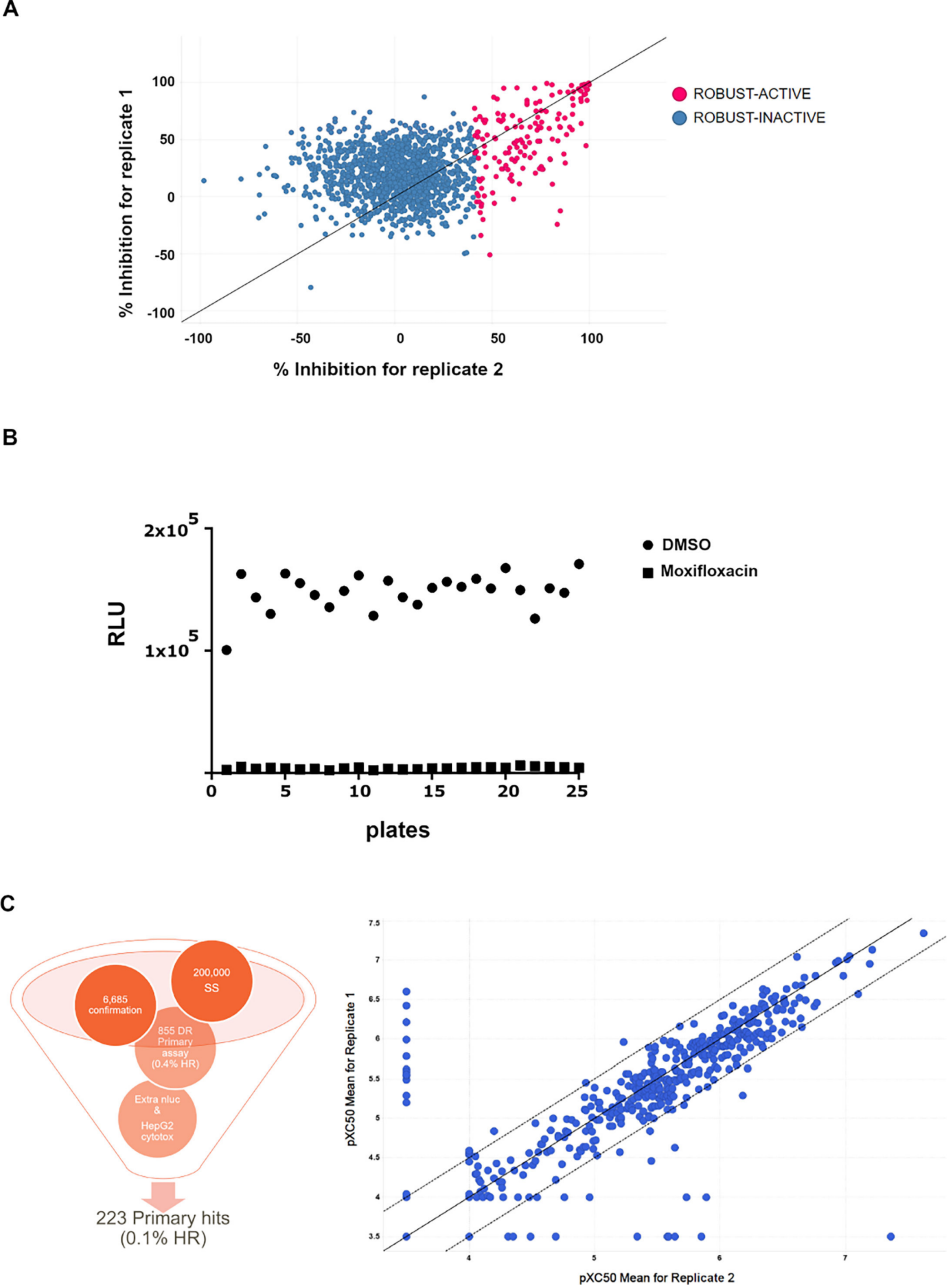
(A) Correlation between replicates of the robustness set. (B) Signal-to-background window in RLU between H37Rv-infected hiPSC-Macs treated with moxifloxacin or DMSO in 25 HTS plates. (C) Summary of the HTS in numbers and dose-response correlation of 855 hits. SS, single shot; DR, dose response; HR, hit rate.

A set of 1,405 compounds formed by a random subset of the entire GSK HTS collection was subjected to a robustness test in Mtb-infected hiPSC-Macs. The aforementioned set was employed to ascertain the suitability of the assay for HTS. The compounds were dispensed in 384-well plates and tested at a final assay concentration of 10 µM for all assays. Duplicates of the robustness set were tested in three independent runs, and a Z-score was calculated for each assay plate to assess signal-to-background variability (Fig. 3A). We found a good correlation between replicates in the same assay and in different runs, with an optimal Z-score (>0.3) for an intracellular assay.

A cut-off rate of 50% inhibition was defined to select compounds as hits. The hits identified were confirmed by dose-response curves.

### High-throughput screen

The HTS campaign was conducted in Mtb-infected hiPSC-Macs with 200,000 compounds, representing the chemical diversity of the GSK collection and excluding potential classical antibiotics by computational methods. The signal-to-background window between DMSO and moxifloxacin, used as controls, is shown for 5% of the HTS (Fig. 3B).

The HTS was run at a single-shot concentration of 10 µM, and 6,685 hits were identified. From those, 855 hits were confirmed by dose-response with a hit rate of 0.4% (Fig. 3C). From 855 initial hits, 369 compounds showed a pIC50 >5 (7.3–5) in Mtb-infected iPSC-Macs (Fig. 3C, right panel). These primary hits were tested in an extracellular assay with the same Mtb reporter strain to identify potential classical antibiotics. From those, at least 100 compounds were classical antibiotics with pIC50 >5 (7.3–5) in the extracellular assay and were then progressed as traditional phenotypic hits. Compounds active in the intracellular assay but inactive in the extracellular assay or with a 10-fold window between *in vitro* activity in hiPSC-Macs and extracellular activity were considered as potential HPI-targeting compounds. Moreover, the cytotoxicity of the primary hits was evaluated in HepG2 cells, and compounds with pIC50 <5 were selected. A selectivity window of 10-fold was established between *in vitro* activity in hiPSC-Macs and cytotoxicity. After these secondary assays, 223 hits possibly related to HPI were selected for further analysis (Fig. 3C, left panel). Table S1 displays the pIC50 values for both intracellular and extracellular assays of the most promising compounds.

## DISCUSSION

Macrophages are central to tissue homeostasis and normal immune response. When they become dysregulated in disease, they are major drivers of inflammation and tissue remodeling in infectious diseases, autoinflammatory disorders, neurodegeneration, and cancer (30). Targeting them at a cellular level is an attractive therapeutic option, as this would impact multiple cellular mediators and functions in parallel. To our knowledge, no marketed drugs target myeloid cells specifically. Yet, recent advances in single-cell technology have revolutionized our understanding of their biology and the subsets associated with the disease. However, a detailed mechanistic understanding of macrophage biology has been hampered by the lack of scalable and/or translationally relevant human models, especially in the context of drug discovery. Conventional approaches rely on immortalized cells, which have limited biological relevance, or primary cells, which are not amenable to large-scale compound or genetic screens. Recent advances in hiPSC-Mac differentiation protocols, including the work described here, provide evidence that hiPSC-Macs retain key functional responses such as phagocytosis, plasticity when polarized with different stimuli, and more faithful correlation to hPBMC-Macs at a transcriptomic level when compared to immortalized cell lines (24–26, 31, 32). Despite the potential of using hiPSC-Macs for drug discovery, very few HTS have been performed to identify new small molecules to date. This is likely due to both the cost of producing hiPSC-Macs and the expertise required to robustly scale up the model. Here, we describe a robust and scalable platform to maintain production of >100 million hiPSC-monocyte-like cells per week, with the resulting macrophages exhibiting >80% expression of canonical surface markers (CD45^+^, CD11b^+^, CD14^+^, CD163^+^, CD86^+^, and CD206^+^) without the need for automation or bioreactor culture. We found that robust and consistent hiPSC-Macs were generated in continuous culture for 12 weeks, and though they can be cultured for longer periods, we observed increased variability in viability and expression of surface markers of the cultures post 12 weeks (personal communication Dr. Lisa Mohamet, GSK). A recent publication by Ackermann et al*.* (21) established an elegant, stirred suspension bioreactor platform that enabled the generation of hiPSC-Macs at a similar scale and timeframe but also included sophisticated sampling of parameters such as oxygen for improved monitoring of the cultures.

The main aim of our work was to employ hiPSC-Macs as a more translationally relevant alternative to immortalized myeloid cells for HTS to identify novel compound series targeting HPI in Mtb. In fact, we have shown that this macrophage model allows us to determine PZA activity with similar results to PBMC-macs. In accordance with this observation, some ESX-1 inhibitors were more active in hiPSC-Macs than in THP1 cells. However, we found that the activity of doramapimod was higher in PBMC-macs and THP1 cells than in iPSC-macs. This could be due to the differential expression of the target involved in different cell types, which will be further explored.

A previous Mtb screening using human embryonic stem cell (hESC)-derived macrophages also demonstrated the identification of compounds targeting HPI (33). Importantly, hiPSCs overcome the ethical concerns of using hESCs (34). In addition, the use of hiPSCs over hESC-derived macrophages enables availability to adult somatic cells from individuals, whereby the donor’s genetic information is retained. This provides an opportunity to identify or select donors based on stratification criteria and the ability to potentially model host polymorphisms that would otherwise be inaccessible, particularly at scale. Furthermore, the use of hiPSCs reduces donor-to-donor variability, likely due to controlled exposure to stimuli that are responsible for immune responses, as observed in cells collected from the blood.

For the first time, we describe an intracellular model of Mtb infection in hiPSC-Macs using a luminescence assay for high-throughput screening of 200,000 unique compounds at a single-shot dose. The Z-score of the screening was above 0.3 in all plates analyzed. This phenotypic screening allows us to expand the repertoire of compounds/targets active against Mtb, including not only compounds targeting HPI but also classical antibiotics, carbon source-dependent compounds, and compounds that accumulate in the macrophages. To distinguish molecules targeting the host or virulence factors, we will perform a battery of secondary assays. Furthermore, target deconvolution will require transcriptomics, proteomics, and other target-based assays to identify potential new modes of action.

## SUPPLEMENTAL MATERIAL

Figure S1 aac.01613-24-s0001.tif Signal-to-background window between the controls in H37Rv-infected hiPSC-Macs (WTSIi018A and UKBi006A) and hPBMC-Macs.

Supplemental legends aac.01613-24-s0002.docx Legends for Fig. S1 and Table S1.

Table S1 aac.01613-24-s0003.docx Intracellular and extracellular activity of 223 hits.

ASM does not own the copyrights to Supplemental Material that may be linked to, or accessed through, an article. The authors have granted ASM a non-exclusive, world-wide license to publish the Supplemental Material files. Please contact the corresponding author directly for reuse.

## References

[1] Chakaya J, Petersen E, Nantanda R, Mungai BN, Migliori GB, Amanullah F, Lungu P, Ntoumi F, Kumarasamy N, Maeurer M, Zumla A. 2022. The WHO Global Tuberculosis 2021 Report - not so good news and turning the tide back to End TB. Int J Infect Dis 124:S26–S29. doi:10.1016/j.ijid.2022.03.011 35321845 PMC8934249

[2] Gries R, Sala C, Rybniker J. 2020. Host-directed therapies and anti-virulence compounds to address anti-microbial resistant tuberculosis infection. Appl Sci (Basel) 10:2688. doi:10.3390/app10082688

[3] Tiberi S, du Plessis N, Walzl G, Vjecha MJ, Rao M, Ntoumi F, Mfinanga S, Kapata N, Mwaba P, McHugh TD, Ippolito G, Migliori GB, Maeurer MJ, Zumla A. 2018. Tuberculosis: progress and advances in development of new drugs, treatment regimens, and host-directed therapies. Lancet Infect Dis 18:e183–e198. doi:10.1016/S1473-3099(18)30110-5 29580819

[4] Huynh J, Donovan J, Phu NH, Nghia HDT, Thuong NTT, Thwaites GE. 2022. Tuberculous meningitis: progress and remaining questions. Lancet Neurol 21:450–464. doi:10.1016/S1474-4422(21)00435-X 35429482

[5] Schutz C, Davis AG, Sossen B, Lai RP-J, Ntsekhe M, Harley YX, Wilkinson RJ. 2018. Corticosteroids as an adjunct to tuberculosis therapy. Expert Rev Respir Med 12:881–891. doi:10.1080/17476348.2018.1515628 30138039 PMC6293474

[6] Young C, Walzl G, Du Plessis N. 2020. Therapeutic host-directed strategies to improve outcome in tuberculosis. Mucosal Immunol 13:190–204. doi:10.1038/s41385-019-0226-5 31772320 PMC7039813

[7] Rybniker J, Chen JM, Sala C, Hartkoorn RC, Vocat A, Benjak A, Boy-Röttger S, Zhang M, Székely R, Greff Z, Orfi L, Szabadkai I, Pató J, Kéri G, Cole ST. 2014. Anticytolytic screen identifies inhibitors of mycobacterial virulence protein secretion. Cell Host Microbe 16:538–548. doi:10.1016/j.chom.2014.09.008 25299337

[8] Tükenmez H, Edström I, Ummanni R, Fick SB, Sundin C, Elofsson M, Larsson C. 2019. Mycobacterium tuberculosis virulence inhibitors discovered by Mycobacterium marinum high-throughput screening. Sci Rep 9:26. doi:10.1038/s41598-018-37176-4 30631100 PMC6328581

[9] Queval CJ, Brosch R, Simeone R. 2017. The macrophage: a disputed fortress in the battle against Mycobacterium tuberculosis. Front Microbiol 8:2284. doi:10.3389/fmicb.2017.02284 29218036 PMC5703847

[10] Ahmed S, Manning A, Flint L, Awasthi D, Ovechkina Y, Parish T. 2022. Identification of novel chemical scaffolds that inhibit the growth of Mycobacterium tuberculosis in macrophages. Front Pharmacol 12:790583. doi:10.3389/fphar.2021.790583 35046812 PMC8762250

[11] Song OR, Deboosere N, Delorme V, Queval CJ, Deloison G, Werkmeister E, Lafont F, Baulard A, Iantomasi R, Brodin P. 2017. Phenotypic assays for Mycobacterium tuberculosis infection. Cytometry A 91:983–994. doi:10.1002/cyto.a.23129 28544095

[12] Sorrentino F, Gonzalez del Rio R, Zheng X, Presa Matilla J, Torres Gomez P, Martinez Hoyos M, Perez Herran ME, Mendoza Losana A, Av-Gay Y. 2016. Development of an intracellular screen for new compounds able to inhibit Mycobacterium tuberculosis growth in human macrophages. Antimicrob Agents Chemother 60:640–645. doi:10.1128/AAC.01920-15 26503663 PMC4704166

[13] Stanley SA, Barczak AK, Silvis MR, Luo SS, Sogi K, Vokes M, Bray M-A, Carpenter AE, Moore CB, Siddiqi N, Rubin EJ, Hung DT. 2014. Identification of host-targeted small molecules that restrict intracellular Mycobacterium tuberculosis growth. PLoS Pathog 10:e1003946. doi:10.1371/journal.ppat.1003946 24586159 PMC3930586

[14] Keiser TL, Purdy GE. 2017. Killing Mycobacterium tuberculosis in vitro: what model systems can teach us. Microbiol Spectr 5. doi:10.1128/microbiolspec.tbtb2-0028-2016 PMC6714986 28597814

[15] Lee CZW, Kozaki T, Ginhoux F. 2018. Studying tissue macrophages in vitro: are iPSC-derived cells the answer? Nat Rev Immunol 18:716–725. doi:10.1038/s41577-018-0054-y 30140052

[16] Tedesco S, De Majo F, Kim J, Trenti A, Trevisi L, Fadini GP, Bolego C, Zandstra PW, Cignarella A, Vitiello L. 2018. Convenience versus biological significance: are PMA-differentiated THP-1 cells a reliable substitute for blood-derived macrophages when studying in vitro polarization? Front Pharmacol 9:71. doi:10.3389/fphar.2018.00071 29520230 PMC5826964

[17] Bernard EM, Fearns A, Bussi C, Santucci P, Peddie CJ, Lai RJ, Collinson LM, Gutierrez MG. 2020. M. tuberculosis infection of human iPSC-derived macrophages reveals complex membrane dynamics during xenophagy evasion. J Cell Sci 134:jcs252973. doi:10.1242/jcs.252973 32938685 PMC7710011

[18] Neehus A-L, Lam J, Haake K, Merkert S, Schmidt N, Mucci A, Ackermann M, Schubert M, Happle C, Kühnel MP, Blank P, Philipp F, Goethe R, Jonigk D, Martin U, Kalinke U, Baumann U, Schambach A, Roesler J, Lachmann N. 2018. Impaired IFNγ-signaling and Mycobacterial clearance in IFNγR1-deficient human iPSC-derived macrophages. Stem Cell Reports 10:7–16. doi:10.1016/j.stemcr.2017.11.011 29249666 PMC5768914

[19] Takata K, Kozaki T, Lee CZW, Thion MS, Otsuka M, Lim S, Utami KH, Fidan K, Park DS, Malleret B, et al.. 2017. Induced-pluripotent-stem-cell-derived primitive macrophages provide a platform for modeling tissue-resident macrophage differentiation and function. Immunity 47:183–198. doi:10.1016/j.immuni.2017.06.017 28723550

[20] Takata K, Kozaki T, Lee CZW, Thion MS, Otsuka M, Lim S, Utami KH, Fidan K, Park DS, Malleret B, et al.. 2020. Induced-pluripotent-stem-cell-derived primitive macrophages provide a platform for modeling tissue-resident macrophage differentiation and function. Immunity 52:417–418. doi:10.1016/j.immuni.2020.01.004 32075730

[21] Ackermann M, Rafiei Hashtchin A, Manstein F, Carvalho Oliveira M, Kempf H, Zweigerdt R, Lachmann N. 2022. Continuous human iPSC-macrophage mass production by suspension culture in stirred tank bioreactors. Nat Protoc 17:513–539. doi:10.1038/s41596-021-00654-7 35039668 PMC7612500

[22] Buchrieser J, James W, Moore MD. 2017. Human induced pluripotent stem cell-derived macrophages share ontogeny with MYB-independent tissue-resident macrophages. Stem Cell Reports 8:334–345. doi:10.1016/j.stemcr.2016.12.020 28111278 PMC5312255

[23] Nenasheva T, Gerasimova T, Serdyuk Y, Grigor’eva E, Kosmiadi G, Nikolaev A, Dashinimaev E, Lyadova I. 2020. Macrophages derived from human induced pluripotent stem cells are low-activated “Naïve-Like” cells capable of restricting mycobacteria growth. Front Immunol 11:1016. doi:10.3389/fimmu.2020.01016 32582159 PMC7287118

[24] Alasoo K, Martinez FO, Hale C, Gordon S, Powrie F, Dougan G, Mukhopadhyay S, Gaffney DJ. 2015. Transcriptional profiling of macrophages derived from monocytes and iPS cells identifies a conserved response to LPS and novel alternative transcription. Sci Rep 5:12524. doi:10.1038/srep12524 26224331 PMC4519778

[25] Vaughan-Jackson A, Stodolak S, Ebrahimi KH, Browne C, Reardon PK, Pires E, Gilbert-Jaramillo J, Cowley SA, James WS. 2021. Differentiation of human induced pluripotent stem cells to authentic macrophages using a defined, serum-free, open-source medium. Stem Cell Reports 16:1735–1748. doi:10.1016/j.stemcr.2021.05.018 34171284 PMC8282471

[26] Zhang H, Xue C, Shah R, Bermingham K, Hinkle CC, Li W, Rodrigues A, Tabita-Martinez J, Millar JS, Cuchel M, Pashos EE, Liu Y, Yan R, Yang W, Gosai SJ, VanDorn D, Chou ST, Gregory BD, Morrisey EE, Li M, Rader DJ, Reilly MP. 2015. Functional analysis and transcriptomic profiling of iPSC-derived macrophages and their application in modeling Mendelian disease. Circ Res 117:17–28. doi:10.1161/CIRCRESAHA.117.305860 25904599 PMC4565503

[27] Zhang JH, Chung TD, Oldenburg KR. 1999. A simple statistical parameter for use in evaluation and validation of high throughput screening assays. J Biomol Screen 4:67–73. doi:10.1177/108705719900400206 10838414

[28] van Wilgenburg B, Browne C, Vowles J, Cowley SA. 2013. Efficient, long term production of monocyte-derived macrophages from human pluripotent stem cells under partly-defined and fully-defined conditions. PLoS One 8:e71098. doi:10.1371/journal.pone.0071098 23951090 PMC3741356

[29] Hall MP, Unch J, Binkowski BF, Valley MP, Butler BL, Wood MG, Otto P, Zimmerman K, Vidugiris G, Machleidt T, Robers MB, Benink HA, Eggers CT, Slater MR, Meisenheimer PL, Klaubert DH, Fan F, Encell LP, Wood KV. 2012. Engineered luciferase reporter from a deep sea shrimp utilizing a novel imidazopyrazinone substrate. ACS Chem Biol 7:1848–1857. doi:10.1021/cb3002478 22894855 PMC3501149

[30] Park MD, Silvin A, Ginhoux F, Merad M. 2022. Macrophages in health and disease. Cell 185:4259–4279. doi:10.1016/j.cell.2022.10.007 36368305 PMC9908006

[31] Liu Y, Li H, Czajkowsky DM, Shao Z. 2021. Monocytic THP-1 cells diverge significantly from their primary counterparts: a comparative examination of the chromosomal conformations and transcriptomes. Hereditas 158:43. doi:10.1186/s41065-021-00205-w 34740370 PMC8569982

[32] Rajab N, Angel PW, Deng Y, Gu J, Jameson V, Kurowska-Stolarska M, Milling S, Pacheco CM, Rutar M, Laslett AL, Lê Cao K-A, Choi J, Wells CA. 2021. An integrated analysis of human myeloid cells identifies gaps in in vitro models of in vivo biology. Stem Cell Reports 16:1629–1643. doi:10.1016/j.stemcr.2021.04.010 33989517 PMC8190595

[33] Han HW, Seo HH, Jo HY, Han HJ, Falcao VCA, Delorme V, Heo J, Shum D, Choi JH, Lee JM, Lee SH, Heo HR, Hong SH, Park MH, Thimmulappa RK, Kim JH. 2019. Drug discovery platform targeting M. tuberculosis with human embryonic stem cell-derived macrophages. Stem Cell Reports 13:980–991. doi:10.1016/j.stemcr.2019.10.002 31680058 PMC6915848

[34] Volarevic V, Markovic BS, Gazdic M, Volarevic A, Jovicic N, Arsenijevic N, Armstrong L, Djonov V, Lako M, Stojkovic M. 2018. Ethical and safety issues of stem cell-based therapy. Int J Med Sci 15:36–45. doi:10.7150/ijms.21666 29333086 PMC5765738

