# Supplementary figures and images for: High-throughput screening of small molecules targeting Mycobacterium tuberculosis in human iPSC macrophages
Source: Antimicrob Agents Chemother. 2025 May 27;69(7):e01613-24. doi: 10.1128/aac.01613-24 (PMC12217486; doi:10.1128/aac.01613-24)

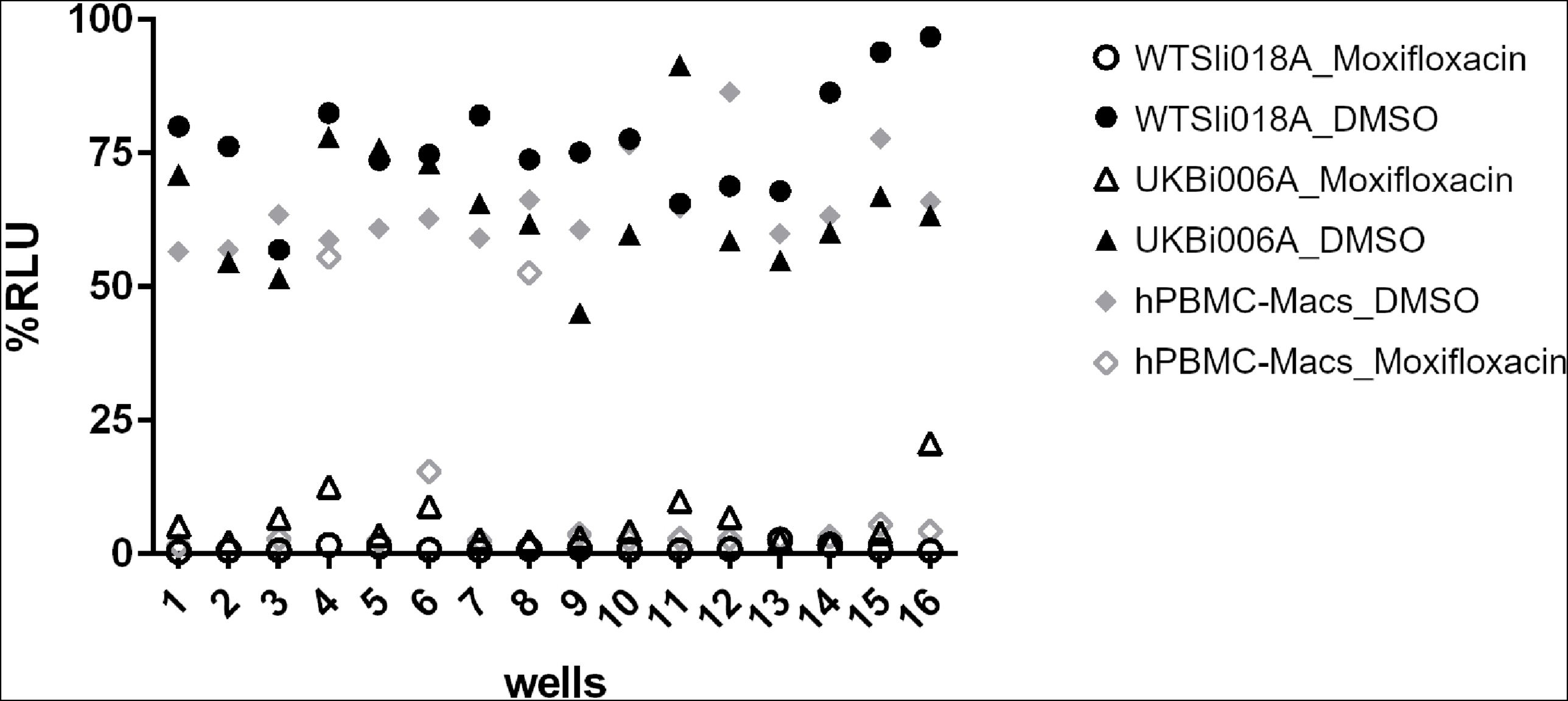

Supplement: Figure S1 — Signal-to-background window between the controls in H37Rv-infected hiPSC-Macs (WTSIi018A and UKBi006A) and hPBMC-Macs. [file aac.01613-24-s0001.tif]
