# Supplementary material for: High-throughput screening of small molecules targeting Mycobacterium tuberculosis in human iPSC macrophages
Source: Antimicrob Agents Chemother. 2025 May 27;69(7):e01613-24. doi: 10.1128/aac.01613-24 (PMC12217486; doi:10.1128/aac.01613-24)
Supplement: Supplemental legends — Legends for Fig. S1 and Table S1. [file aac.01613-24-s0002.docx]

Supplementary Figure 1. Signal-to-background window in the percentage of Relative Light Units (RLU) between H37Rv-infected hiPSC-Macs (WTSIi018A and UKBi006A) and hPBMC-Macs, treated with moxifloxacin or DMSO in 16 wells in two independent assays.

Supplementary Table 1. Activity in H37Rv-infected hiPSC-Macs and extracellular activity of 223 hits possibly related to HPI identified in the HTS. Values are mean pIC50.
