# Supplementary material for: High-throughput screening of small molecules targeting Mycobacterium tuberculosis in human iPSC macrophages
Source: Antimicrob Agents Chemother. 2025 May 27;69(7):e01613-24. doi: 10.1128/aac.01613-24 (PMC12217486; doi:10.1128/aac.01613-24)
Supplement: Table S1 — Intracellular and extracellular activity of 223 hits. [file aac.01613-24-s0003.docx]

Supplementary Table S1. Activity in H37Rv-infected hiPSC-Macs and extracellular activity of 223 hits possibly related to HPI identified in the HTS. Values are mean pIC50.

| **Compound** | **hiPSC-Macs pIC50** | **H37Rv-NLuc pIC50** |
| --- | --- | --- |
| 1 | 5.45 | <4 |
| 2 | 5.19 | <4 |
| 3 | 5.67 | <4 |
| 4 | 5.83 | 4.29 |
| 5 | 5.44 | <4 |
| 6 | 6.12 | <4 |
| 7 | 6.33 | <4 |
| 8 | 6.18 | <4 |
| 9 | 5.66 | <4 |
| 10 | 6.93 | <4 |
| 11 | 6.34 | 4.16 |
| 12 | 5.8 | 4.23 |
| 13 | 5.53 | 4.34 |
| 14 | 6.02 | <4 |
| 15 | 5.19 | <4 |
| 16 | 5.21 | <4 |
| 17 | 5.95 | <4 |
| 18 | 5.05 | 4 |
| 19 | 6.11 | 4.14 |
| 20 | 5.54 | <4 |
| 21 | 6.16 | <4 |
| 22 | 6.49 | <4 |
| 23 | 5.19 | <4 |
| 24 | 5.69 | <4 |
| 25 | 5.33 | <4 |
| 26 | 5.99 | <4 |
| 27 | 5.34 | 4.34 |
| 28 | 5.38 | <4 |
| 29 | 5.32 | <4 |
| 30 | 5.88 | 4.21 |
| 31 | 5.22 | <4 |
| 32 | 5.62 | 4.24 |
| 33 | 5.29 | <4 |
| 34 | 6.08 | <4 |
| 35 | 5.98 | 4.4 |
| 36 | 6.39 | <4 |
| 37 | 6.43 | <4 |
| 38 | 6.06 | <4 |
| 39 | 6.11 | <4 |
| 40 | 6.55 | 4.09 |
| 41 | 5.97 | <4 |
| 42 | 6 | 4.76 |
| 43 | 5.98 | 4.73 |
| 44 | 5.74 | <4 |
| 45 | 6.19 | <4 |
| 46 | 5.49 | <4 |
| 47 | 5.6 | 4.18 |
| 48 | 5.44 | <4 |
| 49 | 5.77 | <4 |
| 50 | 6.3 | 4.22 |
| 51 | 5.11 | <4 |
| 52 | 6.1 | <4 |
| 53 | 5.81 | <4 |
| 54 | 6.46 | <4 |
| 55 | 5.6 | <4 |
| 56 | 5.92 | 4.79 |
| 57 | 6.64 | 4.33 |
| 58 | 5.84 | <4 |
| 59 | 5.76 | <4 |
| 60 | 5.29 | 4.15 |
| 61 | 5.28 | <4 |
| 62 | 6.05 | <4 |
| 63 | 6.13 | <4 |
| 64 | 6.57 | <4 |
| 65 | 5.21 | <4 |
| 66 | 6.8 | <4 |
| 67 | 6.45 | 5.39 |
| 68 | 5.87 | <4 |
| 69 | 7.13 | <4 |
| 70 | 6.27 | <4 |
| 71 | 5.98 | 4.37 |
| 72 | 5.72 | <4 |
| 73 | 5.24 | <4 |
| 74 | 6.11 | 4.9 |
| 75 | 5.54 | <4 |
| 76 | 6.15 | 4.52 |
| 77 | 6.04 | 4.12 |
| 78 | 5.73 | <4 |
| 79 | 5.68 | <4 |
| 80 | 5.84 | <4 |
| 81 | 5.72 | <4 |
| 82 | 5.69 | 4.68 |
| 83 | 6.32 | 4.68 |
| 84 | 5.84 | <4 |
| 85 | 6.72 | 5.24 |
| 86 | 6.68 | 4.12 |
| 87 | 5.21 | <4 |
| 88 | 6.12 | <4 |
| 89 | 5.32 | <4 |
| 90 | 6.09 | 4.48 |
| 91 | 6.2 | 4.85 |
| 92 | 6.18 | 4.77 |
| 93 | 6.23 | 4.8 |
| 94 | 6.18 | 4.73 |
| 95 | 5.67 | 4.18 |
| 96 | 5.43 | <4 |
| 97 | 5.68 | <4 |
| 98 | 5.6 | 4.41 |
| 99 | 5.9 | <4 |
| 100 | 6.11 | <4 |
| 101 | 6.14 | <4 |
| 102 | 6.5 | <4 |
| 103 | 6.58 | <4 |
| 104 | 5.96 | <4 |
| 105 | 5.85 | <4 |
| 106 | 6.62 | <4 |
| 107 | 5.94 | <4 |
| 108 | 5.69++ | <4 |
| 109 | 6.23 | <4 |
| 110 | 6.6 | 4.95 |
| 111 | 5.37 | <4 |
| 112 | 5.32 | <4 |
| 113 | 5.83 | 4.62 |
| 114 | 6.05 | 4.63 |
| 115 | 5.54 | <4 |
| 116 | 5.59 | <4 |
| 117 | 5.92 | 4.18 |
| 118 | 5.97 | 4.34 |
| 119 | 6.21 | 4 |
| 120 | 5.58 | <4 |
| 121 | 5.7 | <4 |
| 122 | 5.43 | <4 |
| 123 | 5 | <4 |
| 124 | 5.66 | 4.11 |
| 125 | 6.31 | <4 |
| 126 | 5.89 | <4 |
| 127 | 6.47 | <4 |
| 128 | 5.39 | 4.24 |
| 129 | 6.02 | 4.11 |
| 130 | 5.25 | <4 |
| 131 | 6.18 | <4 |
| 132 | 5.79 | 4.17 |
| 133 | 5.79 | <4 |
| 134 | 5.35 | 4.03 |
| 135 | 5.77 | 4.66 |
| 136 | 6.04 | <4 |
| 137 | 6.45 | <4 |
| 138 | 5.79 | <4 |
| 139 | 5.49 | <4 |
| 140 | 6.4 | <4 |
| 141 | 5.93 | <4 |
| 142 | 5.82 | <4 |
| 143 | 6.78 | <4 |
| 144 | 5.37 | 4.04 |
| 145 | 5.26 | <4 |
| 146 | 6.2 | <4 |
| 147 | 6.13 | <4 |
| 148 | 5.55 | <4 |
| 149 | 5.63 | <4 |
| 150 | 5.27 | <4 |
| 151 | 5.31 | <4 |
| 152 | 5.63 | <4 |
| 153 | 5.45 | <4 |
| 154 | 6.24 | 4.54 |
| 155 | 5.68 | 4.63 |
| 156 | 5.87 | <4 |
| 157 | 5.56 | 4.5 |
| 158 | 5.22 | 4.11 |
| 159 | 5.28 | <4 |
| 160 | 5.6 | <4 |
| 161 | 5.72 | 4.11 |
| 162 | 6.73 | <4 |
| 163 | 6.54 | 4.02 |
| 164 | 5.57 | <4 |
| 165 | 6.05 | 4.12 |
| 166 | 5.02 | <4 |
| 167 | 6.38 | <4 |
| 168 | 6.12 | <4 |
| 169 | 5.46 | <4 |
| 170 | 5.6 | <4 |
| 171 | 6.11 | 4.33 |
| 172 | 6.08 | 4.02 |
| 173 | 6.12 | <4 |
| 174 | 6.41 | <4 |
| 175 | 5.6 | <4 |
| 176 | 5.57 | <4 |
| 177 | 6 | <4 |
| 178 | 5.01 | <4 |
| 179 | 6.61 | <4 |
| 180 | 5.75 | 4.45 |
| 181 | 5.92 | <4 |
| 182 | 5.88 | 4.29 |
| 183 | 6.19 | <4 |
| 184 | 5.08 | <4 |
| 185 | 6.43 | 5.23 |
| 186 | 6.73 | 5.4 |
| 187 | 6.59 | 4.99 |
| 188 | 5.29 | <4 |
| 189 | 6 | <4 |
| 190 | 5.55 | <4 |
| 191 | 5.38 | <4 |
| 192 | 5.44 | <4 |
| 193 | 5.58 | <4 |
| 194 | 6.72 | <4 |
| 195 | 5.57 | <4 |
| 196 | 6.29 | <4 |
| 197 | 5.67 | <4 |
| 198 | 6.12 | 4.45 |
| 199 | 6.77 | <4 |
| 200 | 5.35 | <4 |
| 201 | 5.42 | <4 |
| 202 | 5.91 | 4.56 |
| 203 | 6.44 | <4 |
| 204 | 6.09 | <4 |
| 205 | 6.24 | <4 |
| 206 | 5.99 | 4.52 |
| 207 | 5.66 | 4.11 |
| 208 | 5.64 | <4 |
| 209 | 5.83 | <4.48 |
| 210 | 5.59 | 4.07 |
| 211 | 5.79 | <4 |
| 212 | 5.98 | 4.69 |
| 213 | 5.55 | 4.11 |
| 214 | 6.23 | <4 |
| 215 | 5.73 | <4 |
| 216 | 5.87 | <4 |
| 217 | 5.09 | <4 |
| 218 | 5.31 | <4 |
| 219 | 5.45 | 4.22 |
| 220 | 5.92 | <4 |
| 221 | 5.44 | <4 |
| 222 | 5.9 | <4 |
| 223 | 6.15 | <4 |
